# Effects of plant growth regulators on *Orobanche coerulescens* seed germination

**DOI:** 10.1186/s40529-025-00479-7

**Published:** 2025-09-29

**Authors:** Yu-Hsuan Wang, Kuan-Hung Lin, Yu-Hsin Tseng, Cung-I Chen, Chang-Chang Chen, Ching-Wen Wang, Meng-Yuan Huang

**Affiliations:** 1 Department of Life Sciences, National Chung-Hsing University, 145 Xingda Rd., South Dist., Taichung, 40227 Taiwan; 2 Department of Horticulture and Biotechnology, Chinese Culture University, Taipei, 11114 Taiwan; 3 Department of Forestry, National Pingtung University of Science and Technology, Pingtung, Taiwan; 4 National Research Institute of Chinese Medicine, Taipei, 11221 Taiwan; 5 Taiwan Biodiversity Research Institute, Nantou, 55244 Taiwan; 6 Innovation and Development Center of Sustainable Agriculture, National Chung-Hsing University, Taichung, 40227 Taiwan

**Keywords:** Conditioning, Holoparasitic, Seeds, Fluridone, Norflurazon

## Abstract

**Background Orobanche coerulescens:**

, used in traditional medicine and as a nutraceutical, requires external chemical stimuli for seed germination, similar to other Orobanchaceae species. This study aims to explore the effects of plant growth regulators conditioning and non-conditioning on its seed germination. Seeds collected from mature plants parasitizing *A. capillaris* were stored at 4 °C to induce dormancy and sterilized with NaOCl. Seeds were sterilized with NaOCl and Tween 20, and preconditioned on agar with gibberellic acid (GA3), fluridone, norflurazon, or brassinolide, and incubated at 4–18 °C for 3–7 days in darkness. Afterward, seeds were transferred to GR24 or SD H_2_O agar, and incubated at 23 ± 2 °C. Non-conditioned sterilized seeds were directly germinated on agar with various substances and incubated for 180 days.

**Results:**

Seeds exhibited a viability of 39.23% at the fifth day after TTC-staining. Fluridone and norflurazon significantly promote germination during conditioning with SD H_2_O or GR24. The highest germination rate occurred with fluridone-conditioned seeds at 18 °C for 5 days, followed by SD H_2_O stimulation. However, GR24 may act as an inhibitor rather than a stimulant.

**Conclusions:**

These findings enhance our understanding of seed germination in this parasitic species, which can aid in conservation efforts.

## Background

The family Orobanchaceae is the largest family of parasitic plants, consisting of 102 genera and over 2,100 species, presenting autotrophic, hemiparasitic, and holoparasitic lifestyles (Nickrent 2020). *Orobanche coerulescens* Stephan & Willd., infests the roots of *Artemisia capillaris* Thunb. through parasitism, thereby receiving nourishment from host-derived nutrients (Zhang et al. 2020; Li et al. 2021). In addition, holoparasitic plants conserve several of the basic features of autotrophic plants at the genetic, biochemical, and physiological levels, and respond to chemical and tactile signals, volatile organic compounds, light, and/or hormone secretion from hosts (Clarke et al. 2019). *O. coerulescens* populations have drastically declined in recent years along with rapid environmental changes, leading to its classification as an endangered species in Taiwan.

Each *Orobanche* seed capsule contains 500 to 1,000 seeds. Seeds represent a form of physiological dormancy (Westerman et al. 2007; Joel et al. 2013). Therefore, the establishment of a persistent seed bank and efficient artificial control over seed dormancy and germination are essential for *Orobanche* to succeed in natural ecosystems and agroecosystems (Fernández-Aparicio et al. 2016). Casadesus and Munne-Bosch (2021) report that *O. ramosa* seeds have a short-term seed mortality of 4–7% per year, and high seed dormancy is synchronized with the host cycle. Nevertheless, the regulation of dormancy and germination, and the germination requirements of *O. coerulescens* seeds, are still unknown although seeds of other Orobanchaceae genera are reported to require an external chemical stimulus for germination. The synthetic strigolactone analogue GR24, bromo-GR24, and fluoro-GR24 all promote *O. cumana* seed germination (Chen et al. 2021). Preconditioning Orobanchaceae seeds for several days in suitable temperatures is required to render imbibed seeds responsive to germination stimulants and allow germination (Plakhine et al. 2009). Germination in response to hormone stimulants can result in the complex regulation of their biosynthesis and in crosstalk with each other (Bouwmeester et al. 2021).

*Orobanche* species have been used as health foods and supplements, and even as drug tonics, in Asia, Europe, America, and Africa (Shi et al. 2020). In Taiwan, *O. coerulescens* is a traditional herbal medicine with nourishing properties, and is a nutraceutical with tremendous economic potential (Qu et al. 2018). This study aims to investigate the effects of plant growth regulators conditioning for stimulant receptivity of GR24 on the germination rate of *O. coerulescens* seed. We hypothesize that conditioning is a specific early phase that allows imbibed seeds to overcome the physiological stress from an immediate germination stimulus. Our results may not only help us better manage *Orobanche* plants and medicinal applications of *Orobanche* species, but also aid in the conservation of and physiological and ecological mechanisms underlying the spread of *O. coerulescens* in natural ecosystems.

## Methods

### Sample collection

**Fig. 1:**
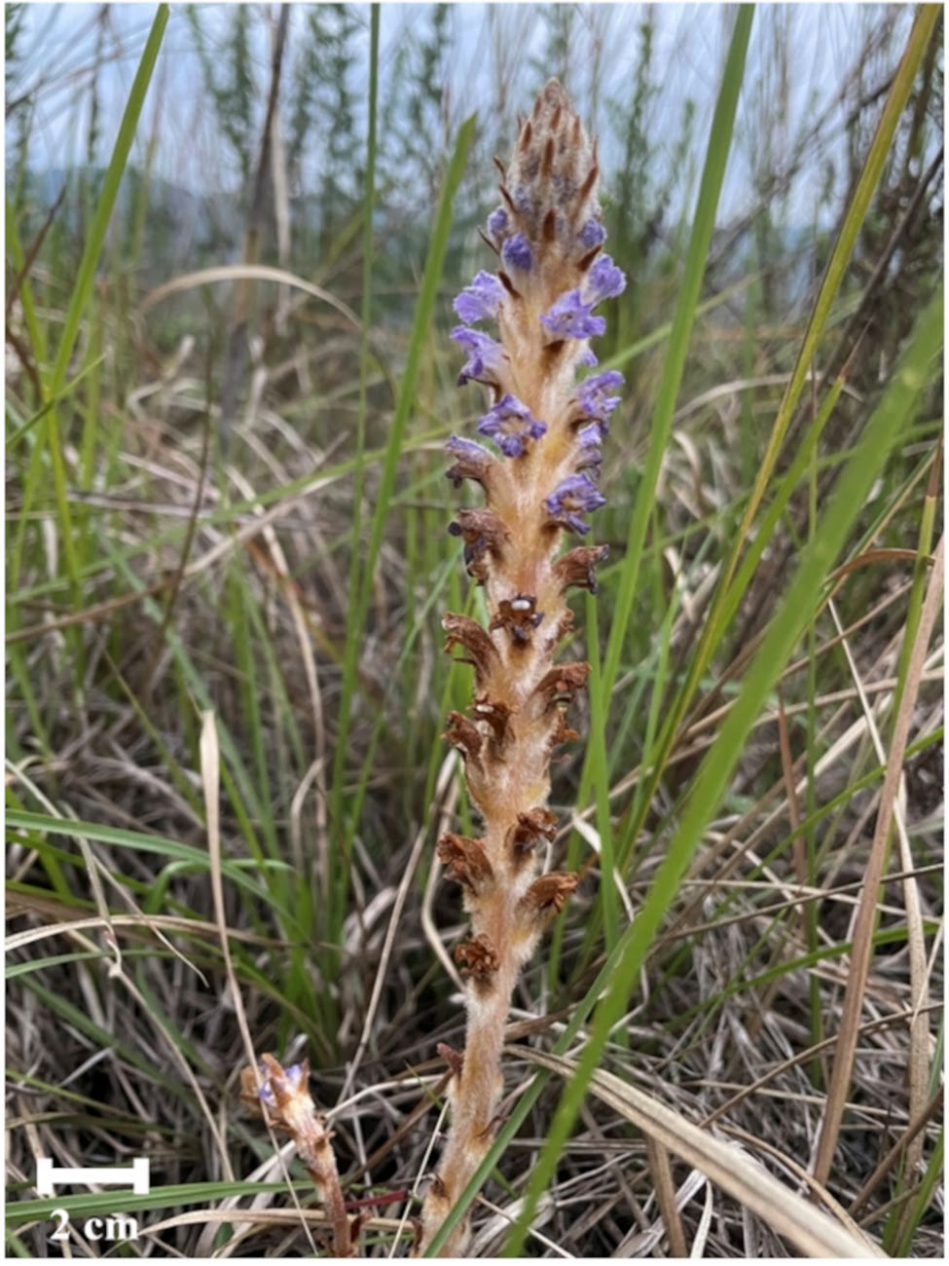
A mature *O. coerulescens* plant parasitizing *Artemisia capillaris* in the Wan-Li riverside field in Hwa-Lien, Taiwan

Seeds of *O. coerulescens* were collected from mature plants that had parasitized *A. capillaris* grown at Wan-Li riverside field in Hwa-Lien, Taiwan, from March to April in 2023 (Fig. 1). Seeds were stored at 4 °C to induce dormancy (Joel et al. 2017).

### Seed viability testing with 2,3,5-triphenyl tetrazolium chloride (TTC)

To assess the influence of fluridone on the viability of *O. coerulescens* seeds, the latter were soaked in 10 mg L^− 1^ of fluridone or sterile deionized water (SD H_2_O, as control), evenly spread on filter paper discs immersed in 2% TTC solution in 9 cm diameter Petri dishes wrapped in aluminum foil, and incubated at 37 °C for 5 days. Seed internal contents staining pink to red indicated they were viable, while unstained seeds indicated non-viability. A stereomicroscope (LEICA MZ6, Wetzlar, Germany) with a 40X objective was used to observe and photograph staining. Fifty fluridone-treated seeds were sprinkled on each filter paper in each Petri dish. There were three replications of each treatment.

### Seed surface sterilization and seed germination

*Orobanche* seeds were surface-sterilized with 0.1%, 0.5%, 0.7%, and 1% NaOCl solutions to evaluate the effect of sodium hypochlorite (NaOCl) treatment during conditioning on seed germination in response to fluridone, each supplemented with 1% Tween 20. Seeds were treated for three minutes, rinsed three times with sterile water, air-dried, and stored in sterilized vials. Subsequently, surface-sterilized seeds were germinated and grown on sterile filter paper in 9 cm diameter Petri dishes in 0.7% (w/v) agar medium (Sigma-Aldrich Co.) containing 10 mg L^− 1^ fluridone, sealed with parafilm, and incubated at 23 ± 2 °C for 180 days in darkness for seed conditioning (Chae et al. 2004; Song et al. 2005). A seed was considered germinated when its radicle protruded from the seed coat, after which it was photographed with a stereomicroscope (LEICA MZ6, Wetzlar, Germany) having a 40X objective. Twenty surface-disinfected seeds were sprinkled onto filter paper in each Petri dish. All treatments received five replicate tests.

### Seed germination assay

#### Seed germination induced without preconditioning plant growth regulators

Seeds were cleaned with 0.1% NaOCl and disinfected for 3 min in 1% (v/v) Tween 20, followed by rinsing with SD H_2_O three times. Subsequently, 20 surface-sterilized seeds were evenly spread over a sterile filter paper in a Petri dish filled with 0.7% (w/v) agar medium. To induce seed germination, 200 mL of GR24 or SD H_2_O were added to the Petri dishes and incubated at 23 ± 2 °C for the designated periods (days) in darkness (Lachia et al. 2015). GR24 was used as a positive control for induced seed germination, while SD H_2_O alone used as a negative control did not induce any germination. Gemination was confirmed by the presence of a radicle protruding from the seed coat using a stereomicroscope with 40X objective. All treatments were tested in five replicates.

#### Seed germination induction with preconditioning plant growth regulators

Seeds were cleaned with 1% (v/v) Tween 20 and disinfected for 3 min in 0.1% NaOCl, followed by rinsing with SD H_2_O three times. Subsequently, 20 surface-sterilized *O. coerulescens* seeds were grown on a sterile filter paper in a Petri dish filled with 0.7% (w/v) agar medium. Surface-sterilized seeds were then preconditioned and germinated on 1% (w/v) agar medium supplemented with 200 L of the following plant growth regulator: 30 mg L^− 1^ gibberellic acid (GA_3_), 10 mg L^− 1^ fluridone [1-methyl-3-phenyl-5-(3-trifluoromethylphenyl)-4-(1 H)-pyridinone], 100 mg L^− 1^ norflurazon [4-chloro-5-methylamino-2-(3-trifluoromethylphenyl) pyridazin-3-one], or 1 mg L^− 1^ brassinolide (Song et al. 2005; Zhang et al. 2009; Joel et al. 2011), and then incubated at 4 and 18 °C for 3 d, 5 d, and 7 d periods in darkness. Plant growth regulator-conditioned seeds after those time periods were transferred to separate Petri dishes filled with 0.7% (w/v) agar medium. Two-hundred mL of GR24 and SD H_2_O were added to Petri dishes and incubated at 23 ± 2 °C in darkness. GR24 and SD H_2_O were used to compare induced germination rates in the preconditioned *O. coerulescens* seeds. All treatments were tested in five replicates.

#### Plant growth regulators application for germination induction without preconditioning

To clarify the effects of the plant growth regulators on the *O. coerulescens* seeds, the seeds were also cleaned by 0.1% NaOCl and disinfected 3 min in 1% (v/v) Tween 20 as described above. Subsequently, 20 surface-sterilized *O. coerulescens* seeds were germinated a on 0.7% (w/v) agar medium supplemented with 200 L of the following plant growth regulators: 30 mg L^− 1^ of GA3, 10 mg L^− 1^ of fluridone, 100 mg L^− 1^ of norflurazon, or 1 mg L^− 1^ of brassinolide, followed by incubated at 23 ± 2 °C in darkness for 180 days. Geminated seeds were observed using a stereomicroscope with 40X objective. All treatments were tested in five replicates. All experiments were repeated five times and found to be reproducible. All chemicals were purchased from Sigma Aldrich (St Louis, MO, USA).

### Statistical analysis

In all experiments, treatments were arranged in a completely random design. Data were subjected to 1-way analysis of variance (ANOVA), with seed viability and germination percentage as factors using Statistical Product and Service Solutions 20 software. Means were compared with the least significant difference (LSD) at the 5% level.

## Results

### Seed viability

**Fig. 2:**
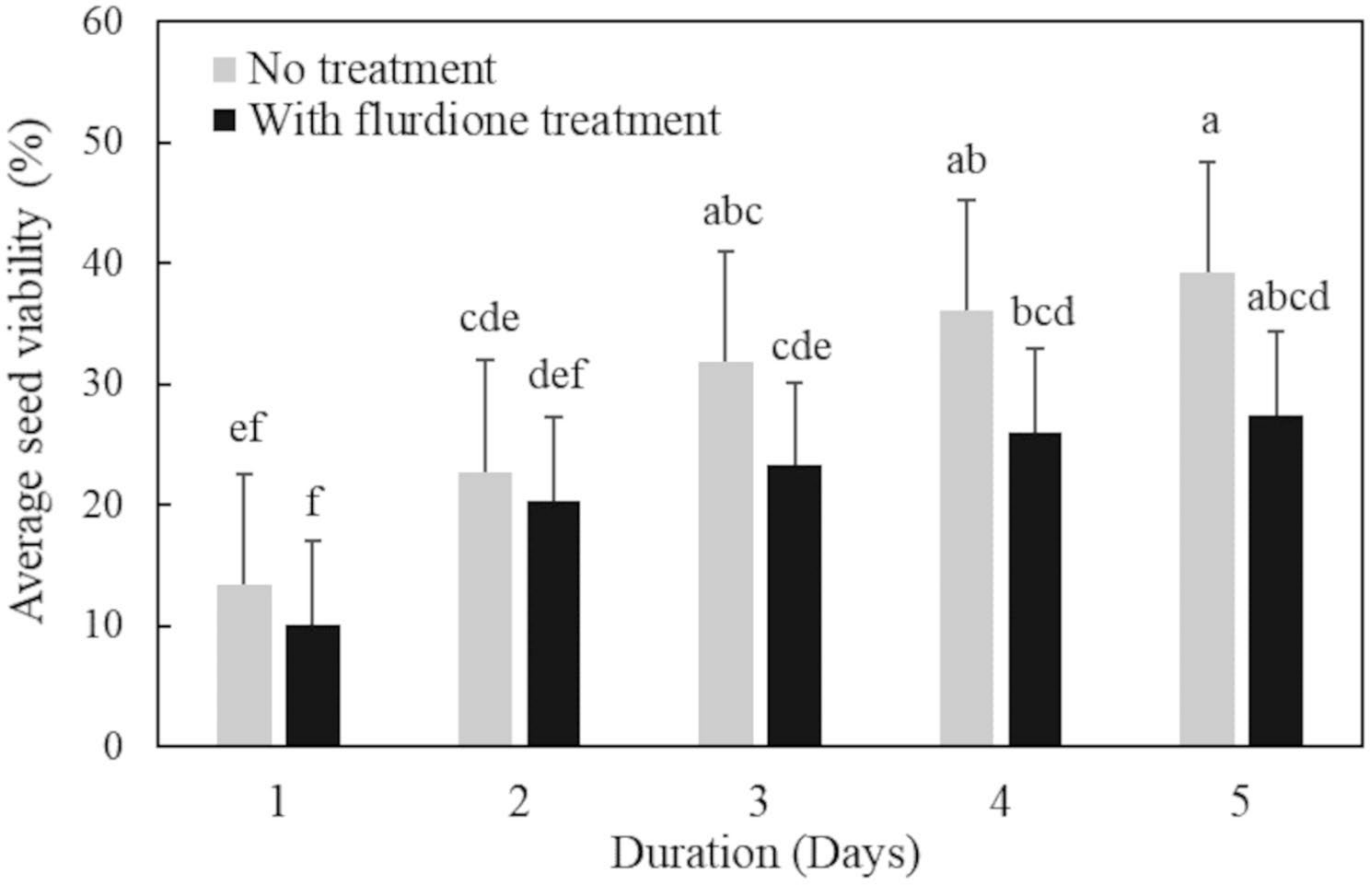
TTC viabilities of *O. coerulescens* seeds with and without fluridone treatment for 5 days. The bar diagram shows the average values with the standard deviation (*n* = 5), and average values with the same letter do not differ significantly according to Fisher’s protected LSD test (*p* < 0.05)

Figure 2 shows the effects of fluridone and SD H_2_O on seed viability. Viability of controls was 13.42% at the first day of TTC testing, and seeds significantly exhibited a viability of 39.23% at the fifth day after TTC staining. However, the highest and lowest TTC viabilities of fluridone-treated seed counts were observed on the first day (10.17%) and fifth day (27.47%).

### Effect of NaOCl treatment on seed germination

**Fig. 3:**
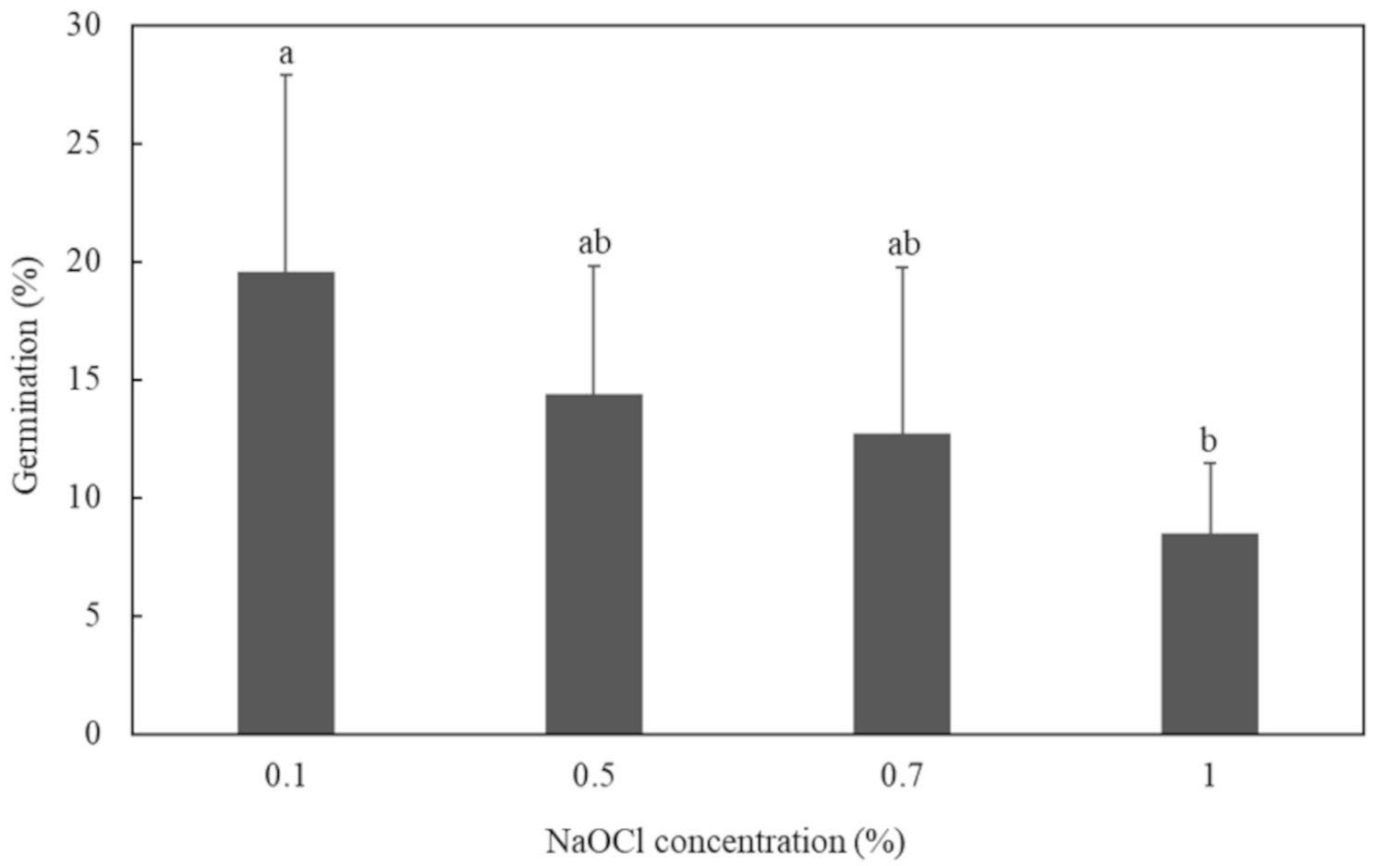
Disinfection effects of NaOCl treatment during conditioning on the germination (%) of *O. coerulescens* seeds in response to fluridone. The bar diagram shows the average values with the standard deviation (*n* = 5), and average values with the same letter do not differ significantly according to Fisher’s protected LSD test (*p* < 0.05)

Figure 3 reveals that disinfected seeds treated with different concentrations of NaOCl have different germination rates. Fluridone effectively increased seed germination in a dose-dependent manner as NaOCl concentrations decreased. Sterilized seeds treated with 0.1% NaOCl displayed a significantly higher germination rate (19.6%) than with 1% NaOCl (8.5%) when fluridone was applied to seeds. Consequently, 0.1% NaOCl treatment was used for the following seed surface sterilization process.

### Effect of plant growth regulators

Seed germination induced without pre-cultivation plant growth regulator application, direct application of GR24 and SD H2O to induce germination in *O. coerulescens* seeds, and no germination was observed after six months of monitoring. Different times, temperatures, and plant growth regulator treatments were tested to determine the optimal conditions for inducing *O. coerulescens* seed gemination. Table 1 shows that seeds conditioned in fluridone, norflurazon, and brassinolide at 4 °C in the dark exhibited various germination rates at different days in response to GR24 under 23 ± 2 °C incubation. Seeds conditioned in the presence of fluridone at the 3rd, 5th, and 7th day during incubation and treated with GR24 displayed 1%, 1.78%, and 1.06% germination rates, respectively. Moreover, norflurazon treatment under GR24 application induced germination rates of 0.67%, 2.22%, and 0.61% at 3, 5, and 7 days, respectively. The highest seed germination rate (3.61%) was found in seeds conditioned in brassinolide at 7 days, while no germination was found in brassinolide at 3 and 5 days. Significantly higher germination occurred in brassinolide treatment (3.61%) than norflurazon treatment (0.61%) at 7 days. However, seeds conditioned in SD H_2_O controls and GA_3_ did not induce any germination in response to GR24.

**Table 1:** Germination (%) of conditioned *O. coerulescens* seeds in response to sterile deionized water (control), GA_3_, fluridone, norflurazon, and Brassinolide at 4 °C/18°C in darkness from day 3 to day 7 of incubation stimulated by GR24/SD water application

	Treatment	Seed germination in conditioning days (%)		
		3	5	7
4 °C GR24	Water	0	0	0
	GA_3_	0	0	0
	Fluridone	1.00 ± 0.91^a^	1.78 ± 2.90^a^	1.06 ± 1.93^ab^
	Norflurazon	0.67 ± 0.91^a^	2.22 ± 3.85^a^	0.61 ± 0.97^b^
	Brassinolide	0	0	3.61 ± 6.90^a^
18 °C GR24	Water	0	0	0
	GA_3_	0	0	0
	Fluridone	2.67 ± 3.03^a^	5.78 ± 9.38^a^	3.00 ± 4.64^ab^
	Norflurazon	1.33 ± 1.39^a^	4.44 ± 5.21^a^	1.56 ± 1.49^b^
	Brassinolide	0	0	6.56 ± 10.63^a^
4 °C SD water	Water	0	0	0
	GA_3_	0	0	0
	Fluridone	0.33 ± 0.75^a^	5.67 ± 7.32^a^	2.67 ± 2.53^a^
	Norflurazon	0.33 ± 0.75^a^	3.33 ± 3.91^a^	2.33 ± 1.90^a^
	Brassinolide	0	0	0
18 °C SD water	Water	0	0	0
	GA_3_	0	0	0
	Fluridone	3.33 ± 3.91^a^	18.00 ± 9.89^a^	12.33 ± 6.52^a^
	Norflurazon	3.00 ± 2.17^a^	5.67 ± 6.08^b^	5.00 ± 6.87^b^
	Brassinolide	0	0	0

Means followed by the same letter within a column under identical temperature and incubation stimulation do not differ significantly (*p* < 0.05) by Fisher’s protected LSD test

Table 1 shows that seeds conditioned in fluridone, norflurazon, brassinolide, SD H_2_O, and GA_3_ at 18 °C have various germination rates in response to GR24. The trend in seed germination from GR24 after hormone conditioning at 18 °C is similar to Table 1 at 4 °C. Seeds conditioned with fluridone at 3, 5, and 7 days and treated with GR24 displayed 2.67%, 5.78%, and 3% germination rates, respectively. Moreover, norflurazon treatment induced germination rates of 1.33%, 4.44%, and 1.56% at 3, 5, and 7 days, respectively. The highest seed germination rate (6.56%) was in seed conditioned in brassinolide at 7 days, which was significantly higher compared to norflurazon treatment (1.56%). Applications of GA_3_ during conditioning greatly inhibited subsequent induced germination rates of *O. coerulescens* seeds.

Table 1 lists the results of seeds being conditioned with various plant growth regulator for different durations at 4 ˚C and 18 ˚C in darkness followed by adding SD H_2_O to induce seed germination. At 4 ˚C, seeds conditioned only with fluridone at 3, 5, and 7 days, then treated with SD H_2_O, displayed 0.33%, 5.76%, and 2.67% germination rates, respectively. Moreover, norflurazon treatment induced germination rates of 0.33%, 3.33%, and 2.33% at 3, 5, and 7 days, respectively. However, seed germination rates in fluridone and norflurazon treatments at 3, 5, and 7 days did not show any significant differences.

The germination trend in seeds subjected to SD H_2_O after plant growth regulator conditioning at 18 °C is similar to that shown for 4 °C. Seeds conditioned with fluridone at 3, 5, and 7 days, and then treated with SD H_2_O, had 3.33%, 18%, and 12.33% germination rates, respectively. Moreover, norflurazon treatment induced germination rates of 3%, 5.67%, and 5% at 3, 5, and 7 days, respectively. Seed germination rates in fluridone treatment at 5 and 7 days were significantly higher than in norflurazon treatment (Table 1).

When plant growth regulators application for germination induction without preconditioning. Furidone and norflurazon were directly applied to seeds after imbibition for 180 days, seeds germination rates of 15.67% and 6.33%, respectively. GA3 and brassinolide did not result in germination.

## Discussion

Estimating seed viability is desirable in seed banking procedures for species of conservation concern. Previously, we tried 0.5 ∼ 2% TTC solutions combined with 25 ∼ 40 ℃ incubation in darkness in seed viability testing, and treatment by staining with 2% TTC at 37 ℃ turned out to be the best method to determine the viability of *O. coerulescens* seeds. Lower seed viability was observed in fluridone-treated seeds (10.17 ∼ 27.47%) than controls (13.42 ∼ 39.23%) tested with TTC. Low seed viability may be a general trait for *O. coerulescens*. Thorogood et al. (2009) reported that Orobanchaceae plant seeds had 38.7–72.6% viability using TTC assays, and GR24 did not induce the germination of all seeds. In our study, seed germinations responsive to fluridone was viable according to TTC viability testing, and the TTC staining rate of seeds (10.17% ∼24.47%) was somewhat higher than their germination rates (8.5 ∼ 19.6%). Fluridone might not induce germination in all viable seeds synchronously at a given time. NaOCl disinfection prior to fluridone treatment is a practical method for improving seed germination. Disinfection is a prerequisite for the in vitro culture of plants, and different sterilizing agents in various concentrations may affect plant survivability. Bar and Mayer (1993) reported that applying hydrogen peroxide during conditioning or during germination significantly reduced germination rates of *Orobanche* seeds. In their study, fluridone induced seed germination rates that gradually decreased as the concentration of NaOCl increased; therefore, 0.1% of NaOCl was recommended for disinfecting *O. coerulescens* seeds.

Seed germination induced without pre-cultivation plant growth regulator application, direct application of GR24 and SD H_2_O to induce germination in *O. coerulescens* seeds, and no germination was observed after six months of monitoring. Therefore, it can be inferred that *O. coerulescens* seeds require different plant growth regulators treatments before the application of GR24 or SD H_2_O in order to successfully induce germination. Under different durations, temperatures, and hormone conditioning, *O. coerulescens* seeds reached maximal germination rates when conditioning in darkness at 18 °C for 5 days with fluridone and norflurazon treatments followed by induction with SD H_2_O and GR24, or when conditioning for 7 days with brassinolide followed by induction with GR24. GR24 stimulation with fluridone and norflurazon preconditioning both attained high germination rates, but it is likely that GR24 was partly restored during the 5-day collection period. Nevertheless, no germination was detected before 7 days after stimulation when brassinolide was applied to conditioned seeds. The duration of exposure to the stimulant apparently affected the germination response pattern of conditioned seeds, and significant germination rates were found after stimulation for 5 days and 7 days at the onset of imbibition. Additionally, the germination rates of fluridone- and norflurazon-conditioned seeds at 4 ˚C in response to SD H_2_O and GR24 stimulations were relatively lower than at 18 ˚C before stimulations. A higher temperature can break seed dormancy, stimulate activity, and initiate germination activities such as absorbing inducing substances and storing germination nutrients. Alternatively, the germination rates of fluridone- and norflurazon-conditioned seeds in response to SD H_2_O stimulation were relatively higher than with GR24 stimulation at all durations, except for the 3-day period at 4 ˚C in response to SD H_2_O. Seeds, preconditioned with fluridone or norflurazon then transferred to water, exhibit a higher germination rate compared to those under GR24. This could indicate that GR24 is an inhibitor rather than a stimulant. All conditioned seeds responded to SD H_2_O and GR24 stimulation, and seed germination was elicited in as soon as 3 days. In fact, conditioned seeds germinated 3–7 days after stimulation, suggesting that the key steps in conditioning take 2–4 days, and the conditioning period needs a specific plant growth regulator related to its biosynthetic pathway that lasts until a germination stimulus is perceived. However, it is still necessary to understand the role of the plant growth regulator-specific conditioning phase. Further studies to examine key physiological activity and regulation during seed conditioning are still needed. Bar and Mayer (1993) report that the respiration peak during the conditioning of *O. aegyptiaca* seeds occurs on day 3, after which the seed is quiescent until stimulated. Plakhine et al. (2009) illustrates that stimulated *O. aegyptiaca* conditioned seeds reach maximal germination rates at 2 weeks after the onset of imbibition. Lechat et al. (2012) reveals that the awakening from seed dormancy of *O. ramosa* requires a minimum of 4 days of conditioning at 21 ˚C for water to enter and imbibition to occur. In our study, when seeds were plant growth regulator-conditioned for 3 ∼ 7 days, seed germination could be induced by SD H_2_O and GR24 treatments.

When fluridone and norflurazon were directly applied to seeds without preconditioning after imbibition for 180 days, seeds reached higher germination rates (15.67% and 6.33%, respectively) than plant growth regulator-preconditioning seeds after SD H_2_O and GR24 stimulations, except that SD H_2_O stimulation took place at 5 days of fluridone conditioning (18%). This further supports the possibility that GR24 is an inhibitor rather than a stimulant. Both fluridone and norflurazone can shorten the conditioning duration required to allow *O. coerulescens* seeds to respond actively to germination stimulants after imbibition. Fluridone and norflurazon are inhibitors of phytoene desaturase catalyzing the desaturation step of phytoene to phytofluene in the carotenoid-biosynthesis pathway, and they also prevent the biosynthesis of abscisic acid (ABA) since carotenoids are the main precursors of ABA (Chae et al. 2004). In addition, fluridone and norflurazon can inhibit ABA biosynthesis and increase germination in *Striga asiatica* seeds (Kusumoto et al. 2006; Jamil et al. 2024). In our study, fluridone and norflurazon induced seed germination when applied at an early stage of conditioning, implying that reducing the ABA content may be crucial for seed germination rather than GR24 stimulation during the carotenoid synthesis pathway. However, seed germination related to ABA inhibition by fluridone and norflurazon, or synthesis of ABA by germinated *O. coerulescens* seeds, needs further investigation.

Lechat et al. (2012) report that *Phelipanche ramosa* seeds require a minimal period of conditioning before stimulant GR24 can trigger germination by breaking ABA dormancy. Song et al. (2005) reports that the germination of *Orobanche* seeds is inhibited when seeds are exposed to GR24 in the early incubation days, but this early GR24-induced inhibition could be reduced by the addition of GA_3_ or norflurazon in the conditioning medium. GA_3_ and brassinolide influence conditioning and germination in *Orobanche* seeds (Takeuchi et al. 1995). GA_3_ biosynthesis occurs during the germination phase of *O. ramosa* seeds (Zehhar et al. 2002). Brassinolide increases cell division and shortens the conditioning period (Takeuchi et al. 1995). However, in our study, GR24 alone and GA_3_-conditioned seeds with GR24 stimulation did not induce the germination of *O. coerulescens* seeds after conditioning for 3 ∼ 7 days with GA_3_ and SD H_2_O. We assumed that 30 mg L^− 1^ of GA_3_ might not be enough for seed conditioning and a response to germination stimulation in *O. coerulescens* seeds. Chen et al. (2016) reports that *C. tubulosa* seeds do not germinate if the ratio of endogenous GA_3_ to ABA is less than 4:3, but exogenous GA_3_ applied to the germination medium does induce seed germination. Studies on different types and concentrations of plant growth regulators to induce the germination of *O. coerulescens* seeds are worthwhile.

## Data Availability

The datasets used and/or analyzed during the current study are available from the corresponding author upon reasonable request.

## References

[CR1] Bouwmeester H, Li C, Thiombiano B, Rahimi M, Dong L (2021) Adaptation of the parasitic plant lifecycle: germination is controlled by essential host signaling molecules. Plant Physiol 185:1292–1308. 10.1093/plphys/kiaa066 33793901 10.1093/plphys/kiaa066 PMC8133609

[CR2] Casadesus A, Munné-Bosch S (2021) Holoparasitic plant–host interactions and their impact on mediterranean ecosystems. Plant Physiol 185:1325–1338. 10.1093/plphys/kiab030 35237829 10.1093/plphys/kiab030 PMC8133675

[CR3] Chae SH, Yoneyama K, Takeuchi Y, Joel DM (2004) Fluridone and norflurazon, carotenoid-biosynthesis inhibitors, promote seed conditioning and germination of the holoparasite *Orobanche minor*. Physiol Plant 120:328–337. 10.1111/j.0031-9317.2004.0243.x 15032868 10.1111/j.0031-9317.2004.0243.x

[CR4] Chen QL, Guo Y, Jiang Y, Tu P (2016) Mechanism of fluridone-induced seed germination of *Cistanche tubulosa*. Pak J Bot 48:971–976

[CR5] Chen Y, Kuang Y, Shi L, Wang X, Fu H, Yang S, Sampietro DA, Huang L, Yuan Y (2021) Synthesis and evaluation of new halogenated GR24 analogs as germination promotors for *Orobanche Cumana*. Front Plant Sci 12:725949. 10.3389/fpls.2021.725949 34603353 10.3389/fpls.2021.725949 PMC8484532

[CR6] Clarke CR, Timko MP, Yoder JI, Axtell MJ, Westwood JH (2019) Molecular dialog between parasitic plants and their hosts. Annu Rev Phytopathol 57:279–299. 10.1146/annurev-phyto-082718-100043 31226021 10.1146/annurev-phyto-082718-100043

[CR12] Fernández-Aparicio M, Flores F, Rubiales D (2016) The effect of *Orobanche crenata* infection severity in Faba bean, field pea, and grass pea productivity. Front Plant Sci 7:1409. 10.3389/fpls.2016.01409 27708660 10.3389/fpls.2016.01409 PMC5030276

[CR7] Jamil M, Alagoz Y, Wang JY, Chen GTE, Berqdar L, Kharbatia NM, Moreno JC, Kuijer HN, Al-Babili S (2024) Abscisic acid inhibits germination of striga seeds and is released by them likely as a rhizospheric signal supporting host infestation. Plant J 117:1305–1316. 10.1111/tpj.16610 38169533 10.1111/tpj.16610

[CR8] Joel DM, Chaudhuri SK, Plakhine D, Ziadna H, Steffens JC (2011) Dehydrocostus lactone is exuded from sunflower roots and stimulates germination of the root parasite *Orobanche Cumana*. Phytochemistry 72:624–634. 10.1016/j.phytochem.2011.01.037 21353686 10.1016/j.phytochem.2011.01.037

[CR9] Joel DM, Gressel J, Musselman LJ (2013) Parasitic orobanchaceae: parasitic mechanisms and control strategies. Springer, Berlin

[CR29] Joel DM, Steffens JC, Matthews DE (2017) Germination of weedy root parasites. In: Kigel J (ed) Seed development and germination. Routledge, New York, pp 567–597

[CR10] Kusumoto D, Chae SH, Mukaida K, Yoneyama K, Yoneyama K, Joel DM, Takeuchi Y (2006) Effects of Fluridone and Norflurazon on conditioning and germination of *Striga Asiatica* seeds. Plant Growth Regul 48:73–78. 10.1007/s10725-005-4746-5

[CR11] Lachia M, Wolf HC, Jung PJM, Screpanti C, De Mesmaeker A (2015) Strigolactam: new potent Strigolactone analogues for the germination of *Orobanche Cumana*. Bioorg Med Chem Lett 25:2184–2188. 10.1016/j.bmcl.2015.03.056 25838142 10.1016/j.bmcl.2015.03.056

[CR13] Lechat MM, Pouvreau JB, Péron T, Gauthier M, Montiel G, Véronési C, Todoroki Y, Le Bizec B, Monteau F, Macherel D, Simier P, Thoiron S, Delavault P (2012) PrCYP707A1, an ABA catabolic gene, is a key component of *Phelipanche Ramosa* seed germination in response to the Strigolactone analogue GR24. J Exp Bot 63:5311–5322. 10.1093/jxb/ers189 22859674 10.1093/jxb/ers189 PMC3431000

[CR14] Li X, Yang JB, Wang H, Song Y, Corlett RT, Yao X, Li DZ, Yu WB (2021) Plastid pseudogenization and gene loss in a recently derived lineage from the largest hemiparasitic plant genus *Pedicularis* (Orobanchaceae). Plant Cell Physiol 62:971–984. 10.1093/pcp/pcab074 34046678 10.1093/pcp/pcab074 PMC8504446

[CR15] Nickrent DL (2020) Parasitic angiosperms: how often and how many? Taxon 69:5–27. 10.1002/tax.12195

[CR16] Nun NB, Mayer AM (1993) Preconditioning and germination of *Orobanche* seeds: respiration and protein synthesis. Phytochemistry 34:39–45. 10.1016/S0031-9422(00)90779-9

[CR18] Plakhine D, Hammam Z, Joel DM (2009) Is seed conditioning essential for *Orobanche* germination? Pest Manag Sci 65:492–496. 10.1002/ps.1716 19222050 10.1002/ps.1716

[CR19] Qu ZY, Jin YP, Zhang YW, Wang ZS (2018) Research advances on chemical constituents, bioactivities and clinical application of medicinal plants in *Orobanche*. Chin J Exp Tradit Med Formulae 24:209–216

[CR20] Shi R, Zhang C, Gong X, Yang M, Ji M, Jiang L, Leonti M, Yao R, Li M (2020) The genus Orobanche as food and medicine: an ethnopharmacological review. J Ethnopharmacol 263:113–154. 10.1016/j.jep.2020.113154 10.1016/j.jep.2020.113154 32763418

[CR21] Song WJ, Zhou WJ, Jin ZL, Cao DD, Joel DM, Takeuchi Y, Yoneyama K (2005) Germination response of *Orobanche* seeds subjected to conditioning temperature, water potential and growth regulator treatments. Weed Res 45:467–476. 10.1111/j.1365-3180.2005.00477.x

[CR22] Song WJ, Zhou WJ, Jin ZL, Zhang D, Yoneyama K, Takeuchi Y, Joel DM (2006) Growth regulators restore germination of *Orobanche* seeds that are conditioned under water stress and suboptimal temperature. Aust J Agr Res 57:1195–1201. 10.1071/AR06131

[CR23] Takeuchi Y, Omigawa Y, Ogasawara M, Yoneyama K, Konnai M, Worsham AD (1995) Effects of brassinosteroids on conditioning and germination of clover Broomrape (*Orobanche minor*) seeds. Plant Growth Regul 16:153–160. 10.1007/BF00029536

[CR24] Thorogood CJ, Rumsey FJ, Hiscock SJ (2009) Seed viability determination in parasitic broomrapes (*Orobanche* and *Phelipanche*) using fluorescein diacetate staining. Weed Res 49:461–468. 10.1111/j.1365-3180.2009.00716.x

[CR25] Westerman PR, Van Ast A, Stomph TJ, Van Der Werf W (2007) Long-term management of the parasitic weed *Striga hermonthica*: strategy evaluation with a population model. Crop Prot 26:219–227. 10.1016/j.cropro.2006.01.017

[CR26] Zehhar N, Ingouff M, Bouya D, Fer A (2002) Possible involvement of gibberellins and ethylene in *Orobanche Ramosa* germination. Weed Res 42:464–469. 10.1046/j.1365-3180.2002.00306.x

[CR28] Zhang R, Xu B, Li J, Zhao Z, Han J, Lei Y, Yang Q, Peng F, Liu ZL (2020) Transit from autotrophism to heterotrophism: sequence variation and evolution of Chloroplast genomes in orobanchaceae species. Front Genet 11:542017. 10.3389/fgene.2020.542017 33133143 10.3389/fgene.2020.542017 PMC7573133

[CR27] Zhang S, Cai Z, Wang X (2009) The primary signaling outputs of brassinosteroids are regulated by abscisic acid signaling. P Natl Acad Sci USA 106:4543–4548. 10.1073/pnas.0900349106 10.1073/pnas.0900349106 PMC2657416 19240210

